# Multi-Day Prolonged Low- to Moderate-Intensity Endurance Exercise Mimics Training Improvements in Metabolic and Oxidative Profiles Without Concurrent Chromosomal Changes in Healthy Adults

**DOI:** 10.3389/fphys.2019.01123

**Published:** 2019-09-03

**Authors:** Dominique D. Gagnon, Sandra Dorman, Stephen Ritchie, Shivaprakash Jagalur Mutt, Ville Stenbäck, Jarosław Walkowiak, Karl-Heinz Herzig

**Affiliations:** ^1^Laboratory of Environmental Exercise Physiology, School of Human Kinetics, Laurentian University, Sudbury, ON, Canada; ^2^Center of Research in Occupational Safety and Health, Laurentian University, Sudbury, ON, Canada; ^3^Northern Ontario School of Medicine, Sudbury, ON, Canada; ^4^Research Unit of Biomedicine, Department of Physiology and Biocenter of Oulu, University of Oulu, Oulu, Finland; ^5^Medical Research Center, Oulu University Hospital and University of Oulu, Oulu, Finland; ^6^Department of Gastroenterology and Metabolism, Poznan University of Medical Sciences, Poznań, Poland

**Keywords:** prolonged exercise, oxidative stress, metabolism, telomeres, hormones

## Abstract

**Background:**

Oxidative stress results in lipid, protein, and DNA oxidation, resulting in telomere erosion, chromosomal damage, and accelerated cellular aging. Training promotes healthy metabolic and oxidative profiles whereas the effects of multi-day, prolonged, and continuous exercise are unknown. This study investigated the effects of multi-day prolonged exercise on metabolic and oxidative stress as well as telomere integrity in healthy adults.

**Methods:**

Fifteen participants performed a 14-day, 260-km, wilderness canoeing expedition (12 males) (EXP) (24 ± 7 years, 72 ± 6 kg, 178 ± 8.0 cm, 18.4 ± 8.4% BF, 47.5 ± 9.3 mlO_2_ kg^–1^ min^–1^), requiring 6–9 h of low- to moderate-intensity exercise daily. Ten controls participated locally (seven males) (CON) (31 ± 11 years, 72 ± 15 kg, 174 ± 10 cm, 22.8 ± 10.0% BF, 47.1 ± 9.0 mlO_2_ kg^–1^ min^–1^). Blood plasma, serum, and mononuclear cells were sampled before and after the expedition to assess hormonal, metabolic, and oxidative changes.

**Results:**

Serum cholesterol, high- and low-density lipoprotein, testosterone, insulin, sodium, potassium, urea, and chloride concentrations were not different between groups, whereas triglycerides, glucose, and creatinine levels were lower following the expedition (*p* < 0.001). Malondialdehyde and relative telomere length (TL) were unaffected (EXP: 4.2 ± 1.3 vs. CON: 4.1 ± 0.7 μM; *p* > 0.05; EXP: 1.00 ± 0.48 vs. CON: 0.89 ± 0.28 TS ratio; *p* = 0.77, respectively); however, superoxidase dismutase activity was greater in the expedition group (3.1 ± 0.4 vs. 0.8 ± 0.5 U ml^–1^; *p* < 0.001).

**Conclusion:**

These results indicate a modest improvement in metabolic and oxidative profiles with increased superoxidase dismutase levels, suggesting an antioxidative response to counteract the exercise-associated production of free radicals and reactive oxygen species during prolonged exercise, mimicking the effects from long-term training. Although improved antioxidant activity may lead to increased TL, the present exercise stimulus was insufficient to promote a positive cellular aging profile with concordant chromosomal changes in our healthy and young participants.

## Introduction

Oxidative stress is the result of an imbalance between the production of free radicals (reactive oxygen species and nitrogen oxygen species), and a defense system of enzymatic and non-enzymatic antioxidants. This imbalance may cause lipid peroxidation (PEROX), as well as protein and DNA oxidation (Urso and Clarkson, 2003; Vollaard et al., 2005; Finaud et al., 2006), leading to metabolic and vascular dysfunctions, cancer, neurological diseases, and even cellular senescence (Xie and Huang, 2003; Cherkas et al., 2008; Puterman et al., 2010; Shammas, 2011; Denham et al., 2013). Mitochondria, at the complexes I, III, and IV, are believed to be a critical source of radicals (estimation of 0.15–5% of oxygen used is reduced to superoxide, hydrogen peroxide, and hydroxyl radicals, among others), along with nicotinamide adenine dinucleotide phosphate (NADPH) oxidase, phospholipase A_2_ (PLA_2_), and xanthine oxidase (Boveris and Chance, 1973; Ashton et al., 1998; St-Pierre et al., 2002; Powers et al., 2011).

Although the production of free radicals is known to originate from multiple organs such as the heart, lungs, and even blood (Powers and Jackson, 2008; Nikolaidis and Jamurtas, 2009), an increase in skeletal muscle activity, and its corresponding response to radical production, has led to many studies and reviews examining the effects of exercise on oxidative stress and health and performance (Vollaard et al., 2005; Finaud et al., 2006; Powers et al., 2011, 2016). While acute exercise, and the concurrent rise in skeletal muscle and mitochondrial activity, increases free radical generation, counterintuitively, regular training lowers systemic oxidative stress levels and improves anti-oxidative activity (Radak et al., 2008). Brites et al. (1999) observed an increase in plasma ascorbic and uric acid concentrations, α-tocopherol, and superoxide dismutase (SOD), representing non-enzymatic and enzymatic antioxidant responses; in trained soccer players compared to sedentary controls. A 12-week training study by Miyazaki et al. (2001) further showed that not only antioxidative responses were influenced by training via higher levels of SOD and glutathione peroxidase (GXP), but oxidative stress was also reduced as neutrophilic superoxide anion and thiobarbituric acid reactive substances (TBARS) were lower post training. This was substantiated by Vincent et al. (2006) in older adults following a 6-month training program where lower levels of TBARS and PEROX as biomarkers of lipid peroxidation were shown. This upregulation of antioxidants is likely a key mechanism of the long-term health benefits of regular exercise. Regular physical activity or training is defined with significant resting sessions where the fluctuating redox status is able to initiate intracellular signaling of antioxidant protein expression. While we have a good understanding of oxidative stress under acute and chronic exercise models, its critical role in many neurological, immune, metabolic, and cardiovascular functions required thoughtful considerations for other types of exercise where redox status may be different.

Interestingly, lower oxidative stress, associated with exercise training, has also been linked to longer telomere length (TL) (Cherkas et al., 2008; Puterman et al., 2010; Shammas, 2011; Denham et al., 2013). Telomeres are repetitive, tandem, hexanucleotide DNA sequences that cap the end of chromosomes, protecting the genomic DNA from enzymatic degradation. They are important in cell division and replication, but are also used as a biological marker of cellular aging and senescence (Shin et al., 2008). TL in somatic cells shortens with age and telomeres that become too short are associated with subsequent cell death. Oxidative stress, induced by the inhibition of the antioxidant glutathione in cultured endothelial cells, has demonstrated acceleration in telomere erosion (Kurz et al., 2004). This suggests that regular exercise and its associated enhancement in anti-oxidants is protecting the degradation of the telomeres and mitigating the aging process and/or enhancing the health of individual cells. Denham et al. (2013) showed that endurance athletes had longer TL (T/S ratio) compared to an age- and sex-matched non-trained group. Six-month endurance or interval training interventions also seem to increase telomerase activity along with TL in lymphocytes, granulocytes, and other leukocytes in previously inactive individuals (Werner et al., 2018). Interestingly, Shin et al. (2008) did not observe an increase in TL following 6 months of training, despite greater GXP activity in erythrocytes.

While we have a good understanding of oxidative stress under acute and regular exercise models, given the critical role in neurological, immune, metabolic, and cardiovascular functions, the thorough understanding of the mechanisms of action for other types of exercise, where redox status may be different, require further study; particularly exercise patterns that are more relevant and generalizable to public application and interpretation. This analysis should include the examination of the associated metabolic and endocrine changes to contextualize the potential mechanisms of action.

One type of exercise that has been understudied to date is prolonged exercise across multiple days. Prolonged exercise is the continuous participation in low-to-moderate intensity activity for 6 or more hours daily, for multiple days. Limited research on the effects of prolonged exercise on oxidative stress have been performed despite its importance in occupational settings; for example, military excursions (Nindl et al., 2007), and wildland firefighting typically incur up to 14 days of continuous, low-to-moderate activity, 8–12 h/day or longer during an emergency (Robertson et al., 2016). Cases et al. (2006) examined antioxidant activity and expression in lymphocytes following a 5-day cycling event at moderate exercise intensity. An increase in SOD isoenzymes CuZn-SOD and Mn-SOD by 2.65- and 2.42-fold, respectively, led to the conclusion that cellular adaptation in antioxidant activity was sufficient to counteract potential cellular damage from the 5-day prolonged oxidative stress. In contrast, a 5-day protocol of exhaustive swimming in Wistar rats demonstrated an increased oxidative stress via elevated levels of TBARS by 131% and conjugated dienes (CD) by 74%, combined with decreased levels of SOD, catalase, glutathione peroxide, and glutathione-*S*-transferase (Thirumalai et al., 2011). During a 330-km, multi-day, ultra-marathon race, Mrakic-Sposta et al. (2015) reported an increase in reactive oxygen species and 8-hydroxy-2-deoxyguanosine (DNA damage). Borghini et al. (2015) demonstrated that, although TL was more preserved in endurance-trained athletes, it still was significantly shorter following the same 330-km ultra-marathon race (post 0.86 ± 0.4 vs. pre 1.11 ± 0.34 T/S ratio).

In summary, despite its fundamental influence on cellular senescence and multiple pathologies, oxidative stress and anti-oxidant responses from exercise have been primarily examined following acute bouts and regular training only, with limited considerations for other types of exercise. Multi-day prolonged exercise may have similar total training workloads compared to months of classic endurance training protocols with lower resting times between bouts. Ultra-endurance prolonged events seem to indicate detrimental effects on TL along with greater oxidative stress, whereas chronic training demonstrates ambiguous results on TL with greater antioxidant activity. The impacts of multi-day prolonged exercise on systemic oxidative stress responses are unclear, as are the associated metabolic, endocrine, and aging effects. Since multi-day prolonged endurance exercise can only be performed within the low- to moderate intensity exercise spectrum interspersed with moderate–vigorous bouts, and given that the majority of the population are not athletes, a focus on non-athlete, healthy adults would provide a greater scope of application to the general population. Therefore, the purpose of the present study was to determine if multi-day prolonged endurance exercise in healthy adults: (i) improves oxidative stress profiling; (ii) positively impacts metabolism; and (iii) influences TL.

## Materials and Methods

### Participants

Twenty-five participants took part in the study in the month of June. Fifteen went on a 14-day, 260-km wilderness canoeing expedition, and 10 acted as controls (did not participate in expedition). Each participant was screened with a PAR-Q and a Health Screening form for cardiovascular, respiratory, or other conditions that could be aggravated by prolonged physical activity. None of the participants were on prescribed medications. Mean (±*SD*) characteristics of the participants are presented in Table 1. Peak oxygen consumption (V̇.O_2__peak_), maximal ventilation (*V*_E_), and maximal heart rate (HR_max_) were identified from the highest value achieved during the maximal incremental treadmill test described below. The study was performed according to the Declaration of Helsinki and was approved by the University Research Ethics Board (LUREB #6011074) and all participants provided written informed consent.

**TABLE 1:** Participant’s fitness characteristics (Mean ± *SD*).

	**Control (6M:4F)**	**Expedition (11M:4F)**	***p*-value**
Age (years)	31.1 ± 10.8	23.5 ± 7.4	0.015^∗^
Height (cm)	174 ± 9.7	177.8 ± 8.5	0.375
*A*_D_ (m^2^)	1.86 ± 0.21	1.87 ± 0.12	
V̇.O_2__max_^1^ (mlO_2_. kg.min^–1^)	47.1 ± 9.0	47.5 ± 9.3	0.745
HR_max_ (beats.min^–1^)	186 ± 9	196 ± 10	0.008^∗^
*V*_E__max_ (L.min^–1^)	110 ± 32	118 ± 31	0.534

^1^Highest value from the maximal adapted incremental treadmill test. *A*_*D*_, body surface area = 0.202 * W^0.425^ * H^0.725^ (DuBois, 1916); $\dot{\text{V}}\,{\text{O}}_{2}\,\text{max}$, maximal oxygen consumption; HR_*max*_, maximal heart rate; V_*Emax*_, maximal minute ventilation. ^∗^Age is significantly lower while HR_*max*_ is significantly greater in the expedition vs. the control group (*p* < 0.05).

### Experimental Protocol

All participants (expedition group: EXP, *n* = 15; controls: CON, *n* = 10) arrived to the laboratory site 24 h before and 3 h after the expedition, in a fasted state, between 07:00 and 10:00 am, preceded by a 24-h period without alcohol, caffeine, and tobacco. Fasting blood and saliva samples, as well as anthropometric data, were collected during these visits. Additional saliva samples were collected every 3 days.

The 14-day canoe trip was conducted on the Bloodvein River in northern Manitoba, Canada. The trip was initiated in Red Lake, Ontario, and concluded at Bloodvein Village, Manitoba. Participants paddled, portaged, hiked, swam, and performed other expedition-related tasks for travel and survival in the wilderness over a distance of approximately 260 km and over 14 days. Based on previous work (Shepard, 1987), and the physical activity intensity compendium (Ainsworth et al., 2000), daily energy expenditure during this period was expected to nearly double with exercise intensity ranging from low (camp chores), to moderate (flat water paddling), to high intensity (white water paddling, portaging, rapid swimming).

### Fitness, Health, and Anthropometric Measures

Percentage body fat (%BF) was estimated from the sum of four skinfolds (Durnin and Womersley, 1974; Bloomer and Fisher-Wellman, 2008), whereas body surface area was calculated from height (H) and weight (W) as follows (DuBois, 1916):

$$A_{\text{D}}=0.202\cdot {\text{W}}^{0.425}\cdot {\text{H}}^{0.725}$$

Body mass index (BMI) was calculated as the mass divided by the square of body height. Waist circumference, hip circumference, as well as waist-to-hip ratio (WHR) were also measured and calculated. A hand-grip dynamometer was also used to provide an indirect measure of whole-body strength. Briefly, the subject held the dynamometer in the right hand, with the arm at right angles and the elbow by the side of the body and with the base resting on first metacarpal (heel of palm), while the handle was resting on middle of the four fingers. When ready the subject squeezed the dynamometer with maximum isometric effort, which was maintained for about 3 s with no other body movement was allowed. The participants performed three contractions with maximum efforts, each separated by 1 min. The highest value was then used analysis. Resting mean arterial blood pressure (MAP) was calculated from assessed systolic and diastolic pressures using an automated blood pressure cuff on the arm (HEM-711AC, OMRON Healthcare Inc., Bannockburn, IL, United States). Maximal oxygen consumption was determined via a treadmill test which was initiated at a speed of 5 mph at a grade of 0% for the first 3 min. Subsequently, elevation was increased by 2.5% every 2 min until the participants reached exhaustion. Oxygen consumption (V̇.O_2_), carbon dioxide release (V̇.CO_2_), and minute ventilation (V̇_E_) were assessed using an open-circuit ergospirometer (Vmax, Sensormedics, CA, United States) with a gas-mixing chamber. Before each trial, the gas analyzers were calibrated with air tanks containing 15% O_2_ and 5% CO_2_, respectively, for calibration of volumes, while the flow sensor was calibrated with a 3-L syringe. A properly adjusted one-way Hans-Rudolph valve connected to a breathing tube was used in all tests to collect expired gases. A heart rate monitor (Polar H10, Kempele, Finland) was also used to assess resting heart rate and maximal heart rate during V̇.O_2__peak_ testing.

### Blood Sampling

Push-button blood collection sets (21G) (BD Vacutainer, Franklin Lakes, NJ, United States) positioned in the antecubital vein were used to collect blood samples in 3.5-ml vacuum-sealed serum tubes with silicon coating (BD Vacutainer SST tubes, Franklin Lakes, NJ, United States), in 3-ml K_2_EDTA whole blood tubes (BD Vacutainer Plus Plastic K_2_EDTA tubes, Franklin Lakes, NJ, United States), and 4-ml heparin gel tube specially designed to isolate monocytes (peripheral blood mononuclear cells) (CPT Cell Preparation Tube, BD Vacutainer, Franklin Lakes, NJ, United States). Samples in serum tubes were given 30 min to coagulate as recommended by the manufacturer whereas plasma and CPT tube were centrifuged immediately. Blood samples were centrifuged at 3500 rpm for 10 min (4100 × *g*) followed by isolation of plasma and serum samples in Eppendorf tubes subsequently frozen at −80°C for analyses. Monocyte samples from CPT tubes were immediately centrifuged at 1800 RFC for 15 min. The white cell layer was pipetted into 15 ml conical tube where it was mixed with 15 ml of PBS for cell washing, and centrifuged again at 300 RFC for 15 min. The supernatant was removed and cells were washed again following the same procedure with 10 ml of PBS. Cell pellets were moved to 2 ml Eppendorf tubes, washed again, before supernatant was removed and cells were frozen at −80°C for telomere analyses.

### Saliva Sampling

Salivary samples were collected before, every 3 days during the expedition, and post expedition. Participants put a cotton swab in their mouth for a duration of approximately 1 min and then gently place the swab in a pre-labeled tube (Salivette^®^, Saliva Examination, Germany). Pre and post samples were immediately frozen at −80°C whereas samples collected during the expedition remained sealed and cooled at ∼5–10°C until the last laboratory visit where they were frozen at −80°C (Chen et al., 1992). Samples from days 3, 6, 9, and 12 were collected prior to overnight sleeping following a water mouth rinse. Samples were later analyzed to assess saliva concentrations of cortisol.

### Oxidative Measures

Oxidative stress was assessed via changes in malondialdehyde (MDA) (Nielsen et al., 1997). Plasma MDA was quantified by using the TBARS kit (Cayman Chemical Company, Ann Harbor, MI, United States). The kit uses the thiobarbituric acid (TBA) to react and adduct the MDA to form the MDA-TBA under high temperature (90–100°C) and acidic conditions. Briefly, 100 μl of plasma was mixed with 100 μl of SDS solution and 4 ml color reagent containing TBA, acetic acid, and sodium hydroxide. The samples were boiled in a water bath for 1 h, cooled, and the absorbance was measured at 530 nm using fluorometric plate reader. The concentration of MDA was calculated and expressed as μM. In addition, SOD was analyzed as an antioxidant marker protein (Castro and Freeman, 2001). The plasma total SOD activity (U/ml) was determined by SOD assay kit (Cayman Chemical Company, Ann Arbor, MI, United States) following the manufacturer’s protocol.

### Relative Telomere Length

DNA from leukocyte cell pellets were isolated from whole blood (BD Vacutainer^®^ CPT^TM^) using Nucleospin DNA isolation kit (MACHEREY-NAGEL GmbH & Co. KG, Germany) (Stenbäck et al., 2019). Relative telomere length (RTL) was determined with qPCR using Cawthon’s monochrome multiplex method (Cawthon, 2002, 2009). Briefly, 2 μl of DNA samples were amplified for 40 cycles, using either telomere or β-globin primers, and the FastStart Universal SYBR Green Master reagent (Roche) in 20 μl final reaction volume. Reactions were run using telomere primers and beta-globin (single copy gene) primers on ABI 7300 real-time PCR system (Applied Biosystems, CA, United States) according to the following conditions: for telomere, 95°C for 10 min, 2 cycles of 95°C for 15 s, 49°C for 15 s and 40 cycles of 95°C for 15 s, 60°C for 15 s, 70°C for 1 min, and for β-globin, 95°C for 10 min, 40 cycles of 95°C for 15 s, 60°C for 1 min followed by a dissociation (or melt) curve for PCR product verification. The RTL was calculated using the 2^–(ΔCt1–ΔCt2)^ = 2^–ΔΔCt^, and expressed as T/S ratio (telomere vs. single copy gene).

The primer sequences used for the qPCR amplifications were for telomere, Telg: ACA CTA AGG TTT GGG TTT GGG TTT GGG TTT GGG TTA GTG T, Telc: TGT TAG GTA TCC CTA TCC CTA TCC CTA TCC CTA TCC CTA ACA. The primers for β-globin were forward: CGG CGG CGG GCG GCG CGG GCT GGG CGG CTT CAT CCA CGT TCA CCTT G, reverse: GCC CGG CCC GCC GCG CCC GTC CCG CCG GAG GAG AAG TCT GCC GTT.

### Metabolic Measures

Serum total cholesterol (TCH), high-density lipoprotein (HDL), low-density lipoprotein (LDL), and triglycerides (TGs) provided a metabolic lipid profile of the participants pre and post expedition. Serum lipid profiles including TCH, HDL cholesterol (HDL-C), LDL cholesterol (LDL-C), and TG were measured by enzymatic method (Siemens Healthcare Diagnostics Inc., Erlangen, Germany). Serum glucose concentration was assayed by an enzymatic method involving hexokinase and glucose-6-phosphate dehydrogenase (Siemens Healthcare Diagnostics Inc., Erlangen, Germany). Serum creatinine levels (Crn) were determined with the Jaffe reaction. Serum electrolytes sodium (Na), potassium (K), chloride (CL), and urea measurements were performed using commercial kits (Siemens Healthcare Diagnostics Inc., Erlangen, Germany).

### Endocrine Measures

Serum total testosterone (ECLIA, Roche Diagnostic, Mannheim, Germany) and plasma insulin levels (DIAsource immunoassays, S.A., Nivelles, Belgium) were analyzed using commercial immunoassay assays. The cortisol level was assessed from saliva samples with luminescence immunoassay kit (IBL International GmbH, Hamburg, Germany) according to the manufacturer’s instructions.

### Statistics

Participant’s age, height, V̇.O_2__max_, HR_max_, and V̇_Emax_ were compared between EXP and CON using Student’s *t*-test with a level of significance set at *p* < 0.05. When normality or equal variance test failed, a Mann–Whitney Rank Sum test was performed. Weight, BMI, %BF, waist circumference, hip circumference, WHR, grip strength, resting HR, and resting MAP were analyzed via a two-way repeated measures ANOVA with time (levels: pre and post) and groups (levels: CON and EXP) as factors. Normality was tested via a Shapiro–Wilk test while the equal variance test via a Brown–Forsythe test. A level of significance was set at *p* < 0.05. Saliva cortisol concentrations were analyzed via a two-way repeated measures ANCOVA, with age, gender, and fitness as covariates, to examine changes before, during, and after the expedition in both groups with group (levels: CON and EXP) and time (levels: baseline, 3 days, 6 days, 9 days, 12 days, and post) as factors. Blood serum fasting oxidative (MDA, SOD), endocrine (TES, INS, COR), and metabolic variables (creatinine, urea, glucose, Na^+^, K^+^, Cl^–^) and RTL from isolated blood mononuclear cells were analyzed via one-way ANCOVA analyses, with age, gender, fitness, and baseline values as covariates. Results were analyzed using STATA version 15 (Statacorp LLC, College Station, TX, United States).

## Results

### Fitness, Health, and Anthropometric Variables

Post expedition, participants in the EXP group demonstrated lower weight (−1.4 kg), BMI (−0.7 m^2^), %BF (−1.2%), waist circumference (−3 cm), hip circumference (−7 cm), and resting heart rate (−11 beat min^–1^) while exhibiting greater grip strength (+8 kg) (*p* < 0.01). The CON group had lower HR (−7 beat min^–1^) and grip strength (−12 kg) (*p* < 0.01) (Table 2).

**TABLE 2:** Participant’s anthropometric and health characteristics pre and post expedition in both the control and expedition group (Mean ± *SD*).

	**Control**		**Expedition**	
		
	**Pre**	**Post**	**Pre**	**Post**
Weight (kg)	72 ± 14.5	72.0 ± 6.2	72 ± 6.2	70.6 ± 6.0^∗^
BMI (kg. m^–2^)	24 ± 3.1	23.6 ± 3.1	23 ± 2.4	22.3 ± 2.2^∗^
Body fat^1^ (%)	23 ± 10.0	22.5 ± 9.5	18 ± 8.4	16.8 ± 9.0^∗^
Waist circ. (cm)	80 ± 11.7	80 ± 12	79 ± 3.9	76 ± 4^∗^
Hip circ. (cm)	99 ± 6.2	100 ± 6	98 ± 3.2^†^	91 ± 4^∗^
Waist–hip ratio	0.81 ± 0.08	0.80 ± 0.09	0.81 ± 0.04	0.83 ± 0.05
Grip strength (kg)	83 ± 21	71 ± 18^∗^	92 ± 22^†^	100 ± 19^∗^
HR resting (b. min^–1^)	71 ± 7	64 ± 8^∗^	74 ± 9	63 ± 9^∗^
MAP resting (mmHg)	90 ± 5	92 ± 7	81 ± 13^†^	85 ± 5

*^1^Estimated from skinfolds (Durnin and Womersley, 1974). BMI, body mass index; circ, circumference; HR, heart rate; MAP, mean arterial pressure. ^∗^Significantly different between pre and post (*p* < 0.05). ^†^Significantly different between control and expedition (*p* < 0.05).*

### Oxidative Stress Variables

**FIGURE 1:**
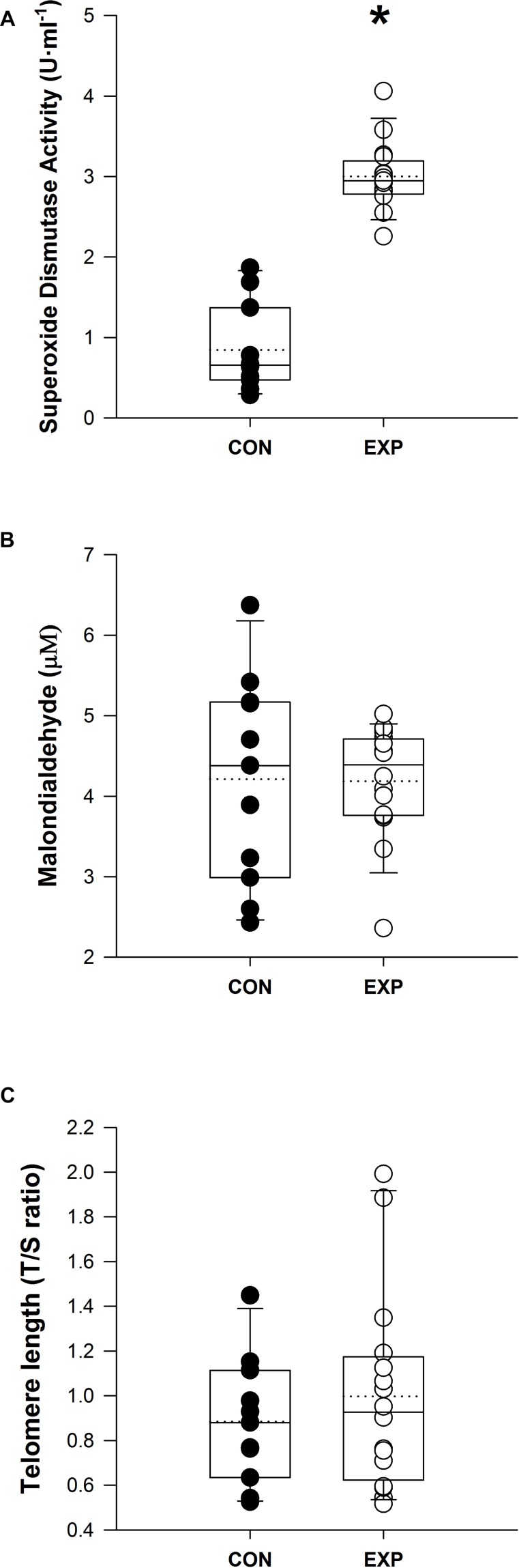
**(A)** Blood plasma superoxide dismutase activity, **(B)** malondialdehyde, and **(C)** mononuclear cells relative telomere length values for each participant in the CON and EXP group. Boxplots line shows median value, dotted line shows the mean, and whiskers show the 5th and 95th percentile. ^∗^Superoxide dismutase is significantly greater in EXP vs. CON.

The enzymatic antioxidant activity from SOD was greater in the EXP group vs. CON [2.9 ± 0.31 vs. 1.1 ± 0.41, *F*(1,20) = 7.127, *p* < 0.001] and was not affected by age (*p* = 0.823), sex (*p* > 0.05), fitness (*p* > 0.05), or baseline values (*p* > 0.05) (Figure 1). Lipid peroxidation from MDA concentrations, however, was not significantly changed between groups (*p* > 0.05) (Figure 1). RTL was also not significantly different between groups (*p* > 0.05) (Figure 1).

### Endocrine Variables

**FIGURE 2:**
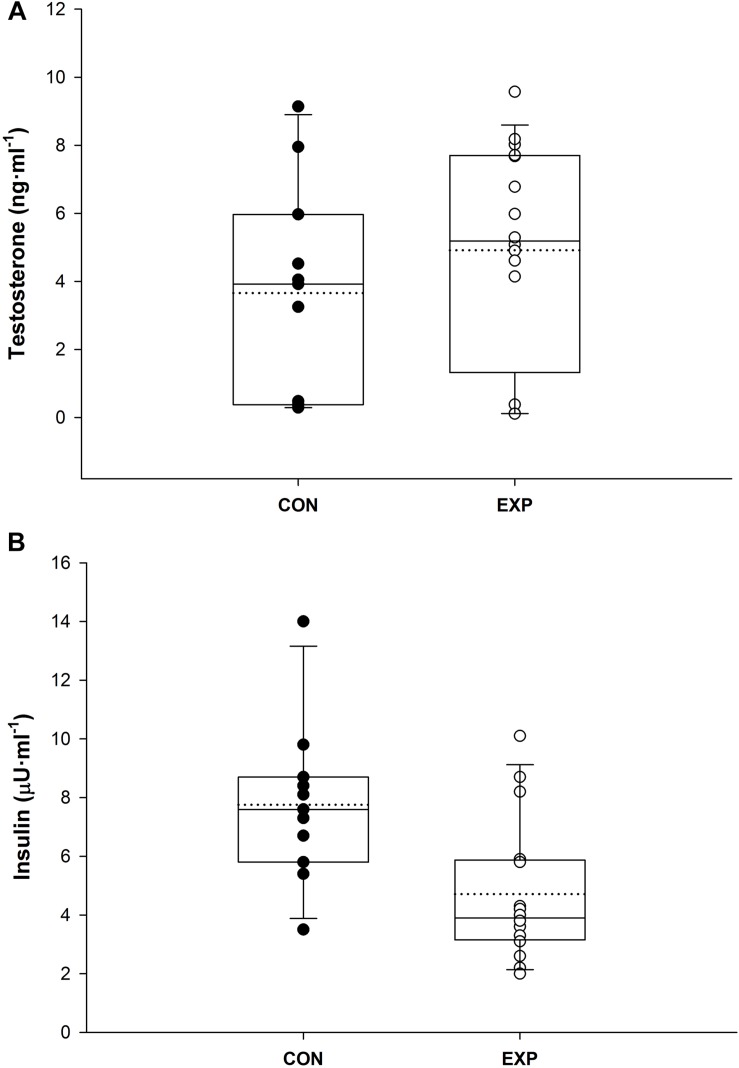
**(A)** Blood plasma testosterone and **(B)** insulin concentration values for each participant in the CON and EXP group. Boxplots line shows median value, dotted line shows the mean, and whiskers show the 5th and 95th percentile.

**FIGURE 3:**
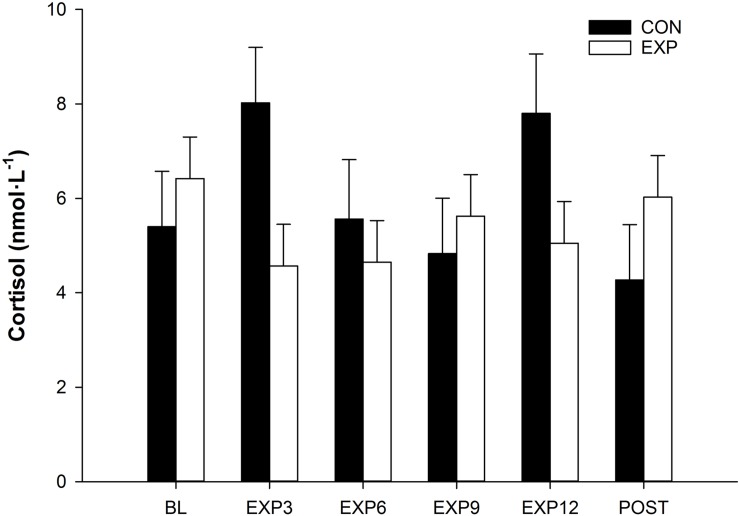
Saliva cortisol concentration values for CON and EXP group at baseline (BL), three in expedition (EXP3), 6 days in expedition (EXP6), 9 days in expedition (EXP9), 12 days in expedition (EXP12), and post expedition (POST). Means ± SEM.

There were no statistical differences between EXP vs. CON for TES (4.7 ± 3.3 vs. 3.65 ± 3.14 ng ml^–1^, *p* > 0.05) (Figure 2). Cortisol also did not yield differences across time (*p* > 0.05) or conditions (*p* > 0.05), nor had any interactions between factors (*p* > 0.05) (Figure 3). However, INS fasting blood concentration was lower in the EXP condition (5.0 ± 2.46 μU ml^–1^) compared to CON (7.8 ± 2.71 μU ml^–1^) [*F*(1,19) = 8.148, *p* < 0.05] and was not affected by any of the covariates (Figure 2).

### Metabolic Variables

Creatinine and glucose were both lower in the EXP group vs. CON [Crn: 0.86 ± 0.022 vs. 1.00 ± 0.025 mg dl^–1^, *F*(1,19) = 16.01, *p* < 0.001; glucose: 86 ± 1.5 vs. 96 ± 1.8 mg dl^–1^, *F*(1,19) = 14.2, *p* < 0.001] and were both influenced by baseline values [creatinine: *F*(1,19) = 24.8, *p* < 0.001; glucose: *F*(1,19) = 17.5, *p* < 0.001] (Table 3). Urea concentration was not different between groups (*p* > 0.05) nor influenced by covariates. Concentrations of Na^+^, K^+^, nor Cl^–^ were not different between EXP and CON (*p* > 0.05) (Table 3).

**TABLE 3:** Metabolic variables.

	**Baseline**		**Post expedition**		
			
	**Control**	**Expedition**	**Control adjusted**	**Expedition adjusted**	
	**Mean ± *SD***	**Mean ± *SD***	**Mean (95% CI U–L)**	**Mean (95% CI U–L)**	***p*-value**
Glucose	101 ± 32	94 ± 5	95(92−100)	86(83−90)	<0.001^∗^
Urea	31 ± 9	31 ± 6	26(22−31)	24(21−28)	0.434
Creatinine	0.93 ± 0.16	0.99 ± 0.13	1.00(0.95−1.05)	0.86(0.81−0.91)	<0.001^∗^
Na+	138 ± 1.5	141 ± 1.8	137(136−139)	138(137−139)	0.678
K+	23 ± 2.2	24.1 ± 1.0	26.0(25−27)	24.8(24−26)	0.132
Cl−	91 ± 3.0	100 ± 4.3	91(88−93)	88(86−90)	0.239

*Na+, sodium (mmol. L^–1^); K+, potassium (mmol. L^–1^); Cl−, chloride (mmol. L^–1^). Glucose, urea, and creatinine are expressed as mg. dL^–1^. ^∗^Glucose and creatinine are significantly lower in the expedition vs. the control group post expedition (*p* < 0.05).*

### Lipid Profile

Blood serum concentrations (Table 4) of CHOL, HDL, and LDL were not statistically different between groups except for TG which was lower in EXP (72 ± 5.1 mg dl^–1^) compared to CON (102 ± 5.9 mg dl^–1^) [*F*(1,19) = 13.307, *p* < 0.01] and was affected by baseline values (*F* = 38.4, *p* < 0.001).

**TABLE 4:** Lipid profile.

	**Baseline**		**Post expedition**		
			
	**Control**	**Expedition**	**Control adjusted**	**Expedition adjusted**	
	**Mean ± *SD***	**Mean ± *SD***	**Mean (95% CI U–L)**	**Mean (95% CI U–L)**	***p*-value**
TC	183 ± 43	167 ± 27	169(158−180)	159(150−169)	0.208
HDL	53 ± 16	54 ± 11	55(50−60)	55(50−59)	0.912
LDL	107 ± 33	95 ± 22	94(85−104)	91(82−99)	0.591
TG	108 ± 66	93 ± 34	102(90−115)	72(61−83)	0.002^∗^

*TC, total cholesterol (mg. dL^–1^); HDL, high-density lipoproteins (mg. dL^–1^); LDL, low-density lipoproteins (mg. dL^–1^); TG, triglycerides (mg. dL^–1^). ^∗^TG is significantly lower in the expedition vs. the control group post expedition (*p* < 0.05).*

## Discussion

This present study examined the influence of multi-day prolonged low- to moderate-intensity endurance exercise on combined oxidative, metabolic, and endocrine profiles as well as telomere integrity in a healthy young human model. Our main findings were that multi-day prolonged low- to moderate-intensity endurance exercise: (1) modestly improved lipid profile and energy metabolism with lower TG, glucose, creatinine, and insulin levels; (2) induced an increase in enzymatic antioxidative activity via greater SOD concentrations; (3) did not have an effect on oxidative stress *per se* as assessed via lipid peroxidation MDA levels; (4) did not result in notable changes in blood testosterone or salivary cortisol levels; and (5) did not result in notable changes in RTL. The expedition group, and not the control group, also had significant decreases in: weight, waist circumference, hip circumference, BMI, and increases in grip strength post-expedition. Altogether, these findings suggest a meaningful, but modest, improvement in both the metabolic and oxidative profiles without an influence on telomere integrity in those participating in multi-day prolonged endurance exercise within, primarily, the low- to moderate-intensity exercise spectrum. In the present study, metabolic improvements were substantiated by lower blood glucose, triglycerides, creatinine, and insulin concentrations as well as lower weight, BMI, and waist and hip circumference.

Although the definition of prolonged exercise is relatively wide, very few reports have examined the effects of exercise interventions beyond multiple days on oxidative stress. Prolonged or intense exercise induces a greater production of free radicals. Increased skeletal muscle activity with associated oxygen demands causes lower O_2_ levels (Richardson et al., 1995) and greater CO_2_ intracellular tension, this combined with an induced lower cellular pH, creates a favorable milieu for oxidative stress at both the mitochondrial and cellular level (Arbogast and Reid, 2004). Vollaard et al. (2005) presented a clear association of acute exercise and increases in TBARS, and lipid oxidation in multiple studies with peak demand of 80 km of running (Kanter et al., 1988). Conversely, training studies, ranging from three to five times per week of exercise for 8–12 weeks, have reported a general improvement of the oxidative profile by both a reduction of free radical production (Leaf et al., 1999; Miyazaki et al., 2001; Radák et al., 2002; Bloomer and Fisher-Wellman, 2008) and an increase in antioxidant activity (Brites et al., 1999; Radák et al., 2002). In the present study, multi-day prolonged low- to moderate-intensity endurance exercise occurred over 14 consecutive days of >7 h of predominantly low-moderate exercise daily. We found under these exercise conditions, oxidative stress was not present, as measured by MDA concentrations; however, there was a significant increase in antioxidant activity as measured via blood concentration of SOD. These findings partly support Cases et al. (2006) with elevated levels of CuZn-SOD and Mn-SOD 2.65- and 2.42-fold. They, however, also noted an increase in carbonyls and reactive oxygen metabolites, both used as oxidative stress markers. Moreover, the endocrine responses in the present study revealed no changes in TES or COR but did detect decreases in serum INS post-expedition in the EXP group only. Prolonged exercise studies have generally demonstrated significant loss in adipose and skeletal muscle mass, as they are generally conducted under hypo-calorific diets, known to diminish TES and increase COR circulating levels (Nindl et al., 2007; Gagnon et al., 2011). We hypothesize that these differences are due to the differences in exercise regimes between groups as well as the study population, notably Nindl et al. (2007) and Gagnon et al. (2011) studies where hypocaloric diets were employed. Our results support this literature, as we also found improvements in health characteristics of our participants, similar to previous reports where exercise loading exceeds a few days (Helge et al., 2006, 2008; Nindl et al., 2007; Gagnon et al., 2011).

### Oxidative Stress

It has been well established that acute exercise induces increases in free radical production stemming from numerous pathways, including anions leaking from the mitochondrial electron transfer chain; due to increased O_2_ consumption and temperature, superoxide production from xanthine oxidase, NADPH oxidase from tissue damage, and auto-oxidation of catecholamines (Deaton and Marlin, 2003). While these processes may be influenced by training with lower free radical production, the increase in anti-oxidative activity from training seems to mitigate the rise in free radicals during exercise more effectively, lowering redox status, and allowing more strenuous physical work to be performed (Leaf et al., 1999). Miyazaki et al. (2001) observed a decrease in neutrophil superoxide anion and TBARS after exhaustive exercise following 12 weeks of running, 5 days per week for 60 min at 80% of VO_2__max_; but no differences in antioxidant enzymes activity. Interestingly, SOD, GXP, and catalase were elevated post training, at rest. A similar finding was obtained by Brites et al. (1999) who found that a group of soccer players had higher levels of antioxidant capacity, ascorbic acid, uric acid, α-tocopherol, and SOD compared to sedentary controls following an overnight fast. In our study, the EXP group arrived at the lab approximately 3 h after exiting the river, providing a resting sample for analyses, and exhibited greater anti-oxidative activity compared to our CON group. Similar to Miyazaki’s training, our results showed no changes in oxidative stress at rest following the exercise intervention, providing some insights on an analogous role of multi-day prolonged exercise compared to long-term training. Cases et al. (2006) reported an increase in both oxidative and anti-oxidative responses after prolonged exercise and oxidative stress but sampling occurred just after by two exhaustive cycling events (164.5 and 166.3 km performed in 255 and 234 min, respectively); highlighting the potential transient nature of these increases. Although the above-mentioned exercise designs contain prolonged exercise, a distinction should be established where our exercise intervention did not lead to physical exhaustion on a daily basis, but rather relied on lower exercise intensities (low and moderate) for a longer duration (6–9 h daily).

#### Telomere Influence

Telomeres contain genomic information necessary for cell preservation and replication. TL and its relationship with cellular senescence has been investigated, along with many influencing factors such as lifestyle, diet, psychological stress, and exercise (Kurz et al., 2004; Cherkas et al., 2008; Puterman et al., 2010; Shammas, 2011). Acute exercise has been shown to induce a measurable decrease in TL in 15 athletes following an ultra-marathon race (Borghini et al., 2015). However, 30 min of running was shown to be sufficient to upregulate 56 miRNAs as well as telomerase-reverse transcriptase (TERT) and sirtuin-6 (SIRT6) mRNA expression from white blood cells; both of these are critical in telomere maintenance and repair (Chilton et al., 2014) suggesting that regular low-to-moderate intensity has beneficial effects on TL. Short bouts of exercise can induce molecular signaling response for increased TL, whereas acute oxidative stress negates, at least briefly, that response. No studies have examined the specific time scale and intensity required to obtain benefits in TL from exercise. There is a gap between our study and others pointing toward unchanged TL even after 6 months of training (Shin et al., 2008), and retrospective work associating TL to long-term physical activity (Kurz et al., 2004; Cherkas et al., 2008; Puterman et al., 2010; Shammas, 2011). Although Werner et al. (2018) indicated that 6 months of endurance (with a total of <60 h of moderate-intensity exercise) or interval training may be sufficient to increase TL. Our study, instead, required approximately 100–120 h of low- to moderate-intensity exercise, which should have been sufficient of a workload to induce a change in TL. While it is difficult to determine what caused the lack of change, intra-individual phenotype and genotype factors may result requiring years of physical activity to observe quantitative and detectable changes (Shin et al., 2008).

### Endocrine and Metabolic Responses

Prolonged physical stress, such as during strenuous exercise/sleep and energy deficit, induces increases in COR, while decreases TES (Nindl et al., 2007; Gagnon et al., 2011). Our measure of oxidative stress, MDA, did not change between groups after the expedition, indicating sufficient rest and food intake during the expedition. These factors limit the influences of stress, and likely explain the lack of differences for those two hormones. Literature highlighting the benefits of being exposed to nature on human health may also contribute to this study’s findings. Beil and Hanes (2013) who examined the effect of visitation to different types of outdoor environments observed lower cortisol levels. Interestingly, we did find changes in the insulin response which was lower in the EXP group. The relationship between oxidative stress, insulin signaling, and resistance has been described (Henriksen et al., 2011; Rains and Jain, 2011). Insulin resistance is induced by increased oxidative stress via the activation of transcription factors (e.g., NF-κB, HIF-1) and the gene expression of pro-inflammatory cytokines (e.g., IL-6, TNF-α), as well as mitochondrial dysfunction (Bloch-Damti and Bashan, 2001; Lowell and Shulman, 2005). Both mechanistic pathways rely on the activation or serine/threonine kinases, which decreases glucose transporter-4 (GLUT-4) expression, and consequently decreases the uptake of glucose into the cell and increase circulating glucose. Helge et al. (2006) examined an increase in GLUT-4 following a 42-day Greenland icecap crossing in the deltoid muscle with no concomitant change in INS circulating levels, nor in insulin sensitivity markers (HOMA, DI, AIR_g_). Interestingly, this was coupled with a decrease in lean body mass and a related decrease in aerobic fitness. Our participants lost a mean 1.4 kg of mass and 1.2% of BF, for a loss of 0.5 kg of lean mass. This is not comparable to the nearly 3 kg of Helge et al. (2006), suggesting that muscle loss is not an important factor in the upregulation of GLUT-4. Houmard et al. (2004) examined exercise volume, duration, and intensity on insulin sensitivity and found that, across the three components, duration had the largest effect on insulin sensitivity. The multi-day prolonged exercise from our intervention could have consequently increased insulin sensitivity, lowering the need for insulin secretion and maintaining a lower glucose level; all indicators of improved metabolic health.

Finally, we also measured a lower level of Crn in this study. It is unlikely that this effect is due to enhanced renal functions and clearance post-expedition; given the age and health of our study population. We hypothesize this effect is due to two changes in metabolism from the activity itself. First, we believe that there was likely an enhanced, continuous uptake of amino acids by the muscles cause a decline in all amino acids including Crn. This is supported by both the measured increase in grip strength as well as our estimated increase in total muscle mass. In addition, under these conditions of prolonged, low-to-moderate intensity exercise, the cells would enhance the protective mechanisms to prevent muscle losses, this would include a metabolic shift toward enhanced lipid metabolism and suppressed protein and carbohydrate metabolism. This hypothesis is supported by the measured decreased in fat tissue and overall weight loss (Horowitz and Klein, 2000). It is well known that endurance training induces a shift toward lipid metabolism. Interestingly, Tunstall et al. (2002) presented a clear activation of lipid metabolism gene expression after only 9 days of endurance training with increases in FAT/CD36, CPT1, PPARα, PPARγ, and others. Our participants were physically active for 14 days, and would have expressed a similar shift.

### Considerations

Some limitations were identified in this study. The weight loss observed following the expedition in the EXP group (−1.4 kg) was minimal but nonetheless demonstrated that multi-day prolonged exercise was performed under hypocaloric conditions, despite our best attempt to maintain an equicaloric diet for our participants. Hypocaloric diets during multi-day prolonged exercise have previously been associated with detrimental health effects (i.e., dysregulated lipids concentrations, muscle mass loss), and a noticeable decrease in circulating total and free TES levels (Nindl et al., 2007; Gagnon et al., 2011). These findings were not present in the current study, suggesting that nutritional intake was sufficient to maintain exercise demands without compromising endocrine and health measures. This is further supported by the improvement in grip strength detected in the EXP group. Furthermore, our data collection platform included MDA as a lipid peroxidation marker for oxidation stress, yet the inclusion of protein (carbonyl and/or carbonyl/protein ratio) as well as DNA (8-hydroxy-deoxyguanosine) oxidative markers would have greatly contributed to understanding of the present results. Similarly, assessing enzymatic antioxidants such as catalase and glutathione as well as non-enzymatic antioxidants as variables would have benefited this study. Moreover, while our groups were composed of both males and females, only three females were part of each group. Even if sex was used as a co-variate within our analyses, critical physiological differences between males and females may limit the present interpretation of some results, particularly in the case of TES concentrations since the effects of exercise were reflected very differently between both the sexes. Finally, we were unable to perform a V̇.O_2__max_ test post expedition, which would have determined whether cardiorespiratory capacities were influenced by the exercise design, and whether a training effect occurred.

### Implications and Perspectives

Many studies have examined the use of antioxidants (e.g., polyphenolics and vitamins E and C) (Morillas-Ruiz et al., 2006) during or following exercise and training, and their effects on muscle damage, inflammation, or the maintenance of physical performance parameters such as skeletal muscle fiber contractile properties, excitation contraction coupling, power output, and fatigue (Alessio et al., 2000; Clarkson and Thompson, 2000; Kubukeli et al., 2002). While this may be useful in an acute exercise setting, the blunting of the increase in free radicals prevents the activation of gene expression signaling pathways of exercise-induced mitochondrial biogenesis and enzymatic antioxidants defense system (Nieman et al., 2010; Petersen et al., 2012). The redox status influences telomere state and not solely the generation of free radicals, and the use of antioxidants which could influence telomere integrity. Future investigations should consider the long-term influences of certain antioxidant supplements on longevity and health.

## Conclusion

The present study offered insight on the oxidative stress response, antioxidant response, metabolic and endocrine changes, as well as telomere influence from exposure to multi-day prolonged low- to mid-intensity endurance exercise, in a healthy, but non-athlete population. Our main findings are that this type of exercise, without exhaustion, modestly improved lipid profile and energy metabolism while increasing enzymatic antioxidative activity, while have no detectable changes in resting endocrine responses or in RTL; mimicking similar redox balance benefits from training ranging from weeks to months.

## Ethics Statement

This study was carried out in accordance with the recommendations of the Canadian Tri-Council research guidelines and the Research Ethics Board of Laurentian University, with written informed consent from all subjects. All subjects gave written informed consent in accordance with the Declaration of Helsinki. The protocol was approved by the Research Ethics Board of Laurentian University.

## Author Contributions

DG, SD, SR, and K-HH designed the study and conducted the experiment. SM, VS, and JW performed the biochemical analyses of blood and saliva samples. All authors offered critical feedback on results and findings and contributed to the writing of the manuscript.

## Conflict of Interest Statement

The authors declare that the research was conducted in the absence of any commercial or financial relationships that could be construed as a potential conflict of interest.
